# Biomarkers for Predicting Left Atrial or Left Atrial Appendage Thrombus in Anticoagulated Patients with Nonvalvular Atrial Fibrillation

**DOI:** 10.1155/2020/1683142

**Published:** 2020-06-20

**Authors:** Xue Zhou, Zuolan Wang, Shuang Dou, Kangyin Chen, Enzhao Liu, Tong Liu, Guangping Li, Jingjin Che

**Affiliations:** ^1^Tianjin Key Laboratory of Ionic-Molecular Function of Cardiovascular Disease, Department of Cardiology, Tianjin Institute of Cardiology, The Second Hospital of Tianjin Medical University, Tianjin 300211, China; ^2^Chaoyang Central Hospital, Chaoyang, Liaoning 122000, China

## Abstract

**Purpose:**

Although atrial fibrillation (AF) is often associated with thromboembolic complications, there is no definite biomarker for detecting the presence of thrombi in the left atrial (LA) or left atrial appendage (LAA) in patients with nonvalvular atrial fibrillation (NVAF).

**Methods:**

NVAF patients who underwent transesophageal echocardiography (TEE) to evaluate LA/LAA thrombus and spontaneous echo contrast (SEC) before AF ablation were included. Multivariate logistic regression and receiver operating characteristic curve (ROC) analyses were performed to explore the independent risk factors of LA/LAA thrombus and indicate the best cutoff point.

**Results:**

Of the 260 consecutive subjects (mean age: 63.67 ± 9.39 years; 42% women), 45 (17.3%) patients were with LA/LAA thrombus, 131 (50.4%) were with SEC, and 84 (32.3%) were with neither thrombus nor SEC. The results of multivariate logistic regression analysis showed that N-terminal pro-B-type natriuretic peptide (NT-proBNP) (OR, 2.179; 95% CI: 1.191–3.987; *p*=0.012) and red cell distribution width (RDW) (OR, 2.398; 95% CI: 1.075–5.349; *p*=0.033) were independently correlated with the presence of LA/LAA thrombus but not D-dimer (OR, 0.999; 95% CI: 0.998–1.000; *p*=0.210). When all patients were divided into four groups based on the combination between RDW (cutoff value: 12.95%) and NT-proBNP levels (cutoff value: 368.9 ng/L), the rate of LA/LAA thrombus was the highest in the high RDW and NT-proBNP group.

**Conclusion:**

In anticoagulation patients with NVAF, elevated NT-proBNP and RDW are related to LA/LAA thrombus. Therefore, these might be considered as useful prognostic markers in the management and treatment of NVAF patients.

## 1. Introduction

Atrial fibrillation (AF) is a common cardiac arrhythmia and is regarded as one of the major causes of stroke, heart failure, sudden death, and cardiovascular morbidity in the world [1]. Furthermore, the number of patients with AF is predicted to steeply rise in the coming years. Due to failed effective and regular atrial contraction, AF patients usually have slow blood flow, blood stasis, and even thrombosis in the left atrium (LA) or left atrial appendage (LAA) [2]. As the thrombus dislodges, it might induce systemic embolism, stroke, etc., seriously affecting the quality of life and threatening the life of patients with AF. Although various types of scoring systems, such as the CHADS_2_ and CHA_2_DS_2_-VASc scores, have been developed for predicting the risk of embolic events in nonvalvular atrial fibrillation (NVAF) patients [3, 4], these offered a modest predictive value for the presence of LA thrombus.

Transesophageal echocardiography (TEE) is a more sensitive approach for detecting the thrombus in LAA. However, it might not be accepted or tolerated by patients due to its disadvantages of trauma to oropharynx, esophagus, or stomach caused by the insertion and operation of ultrasound probe. LAA thrombi and spontaneous echo contrast (SEC) often coexist and can be difficult to differentiate using TEE, particularly when SEC is dense [5]. Therefore, the need for reliable alternative biomarkers such as N-terminal pro-B-type natriuretic peptide (NT-proBNP), D-dimer, and red cell distribution width (RDW) is growing in contemporary clinical practice [6–8].

The hypercoagulable state of AF is mirrored by increased concentrations of a variety of prothrombotic markers, and D-dimer is regarded as the gold standard among these [9]. Therefore, increasing investigation has been conducted to explore the association of D-dimer in patients with AF in the presence of LA thrombosis. But the studies presented to date have presented conflicting results. Both Apixaban for the Prevention of Stroke in Subjects With Atrial Fibrillation (ARISTOTLE) trial and the Randomized Evaluation of Long-Term Anticoagulant Therapy (RE-LY) trial showed that the baseline levels of D-dimer were related to the rate of stroke/systemic embolism. Addition of baseline levels of D-dimer to established clinical risk factors improved the prediction of stroke/systemic embolism [10, 11]. However, You and Tang revealed that only D-dimer levels at stroke onset were considered as independent risk factors of ischemic stroke, while increase in baseline D-dimer levels was not an independent risk factor [12]. D-dimer levels had no predictive value with regard to the occurrence of ischemic stroke in patients with NVAF [13].

In addition, it is speculated that NT-proBNP is of atrial origin in AF patients due to myocyte stress in the atria, which thus reflects atrial dysfunction [14]. ARISTOTLE and RE-LY trials also showed that the levels of NT-proBNP were correlated with high risk of thromboembolic events and cardiovascular mortality [8, 15]. A strong association was found between the elevated risk of ischemic stroke and rising NT-proBNP levels. However, the relationship between NT-proBNP levels and the presence of LA/LAA thrombosis in AF patients needs further clarification.

In recent years, many studies have reported that elevated levels of RDW acts as a strong independent predictor of acute and chronic heart failure, myocardial infarction, atrial fibrillation, and stroke. Erythrocytes are involved in hemostasis, and any abnormalities in the quantity and quality of red blood cells promote the formation of thrombosis in vivo and enhance the stability of thrombosis [16, 17]. In addition, studies showed RDW as an independent predictor of high CHADS_2_ and CHA_2_DS_2_-VASc scores in patients with AF [18].

Biomarkers derived from the blood might help to refine the risk assessment for AF in stroke outcomes and mortality. However, there is no definite biomarker for the presence of thrombi in LA or LAA during anticoagulation in patients with AF. Hence, this study aimed to investigate the correlation of preprocedural D-dimer, RDW, NT-proBNP, and other clinical risk factors in the presence of LA/LAA thrombus during anticoagulation in patients with NVAF.

## 2. Materials and Methods

### 2.1. Patient Selection

Patients with NVAF who underwent TEE for the detection of intracardiac thrombus before AF ablation between December 2012 and December 2018 at the 2nd Hospital of Tianjin Medical University were enrolled. The definition and classification of NVAF used in this study were based on the published guidelines of the American College of Cardiology-American Heart Association and the European Society of Cardiology [19]. All patients undergoing AF ablation should be anticoagulated with the non-vitamin K antagonist oral anticoagulant (NOAC) or warfarin with a therapeutic INR of 2.0–3.0 for 3 weeks prior to the procedure; after the procedure, the patients should receive anticoagulation for at least 2 months [20]. The anticoagulant time of warfarin and NOAC started from INR > 2.0 and oral drug time, respectively. TEE was carried out within 24 h before AF ablation treatment. Patients with valvular atrial fibrillation were excluded from the study. Additionally, patients with venous thrombosis, acute or chronic pulmonary embolism, aortic dissection, existing symptoms of severe heart failure (NYHA class IV), autoimmune disease, acute myocardial infarction, renal failure (eGFR < 30 mL/min/1.73 m^2^), known hepatic impairment (total bilirubin >3 mg/mL), malignancies, and acute or chronic inflammatory diseases were excluded. This study was approved by the Ethics Committee of the 2nd Hospital of Tianjin Medical University. Written informed consent was obtained from all participants.

### 2.2. Clinical Characteristics

In this study, all blood samples from NVAF patients were collected and measured after admission and before TEE examination. The baseline demographic data (age and gender), medical history (hypertension, diabetes mellitus, coronary heart disease, old myocardial infarction, paroxysmal AF, prior stroke or transient ischemic attack (TIA), recurrent AF after ablation, anticoagulant use prior to the reference TEE, laboratory blood biomarkers (erythrocytes markers, coagulation markers, myocardial damage markers, creatinine, urea nitrogen, NT-proBNP, D-dimer, and cholesterol), and echocardiographic parameters were obtained from the clinical records. The stroke risk in these patients was assessed by CHA_2_DS_2_-VASc scores, which was calculated by adding the risk factors of congestive heart failure, hypertension, age 65 to 74 years or ≥75 years, diabetes mellitus, stroke or TIA, vascular disease, and female gender. Each parameter was weighed by “1,” except for stroke or TIA and age ≥75 years that were weighed by “2” [4].

### 2.3. Transesophageal Echocardiography

Although the anticoagulation strategies have been improved, thromboembolism is still a serious complication. TEE is the gold standard examination for LA/LAA thrombus and is performed to minimize the risk of periprocedural thromboembolic events. In this study, TEE was performed 1 or 2 days prior to undergoing catheter ablation. LA and LAA were investigated for the presence of thrombus and SEC in different tomographic planes of TEE. Thrombus in LA/LAA was defined as a well-circumscribed, echogenic mass with a different texture when compared with the LA wall and has uniform consistency and not related to the pectinate muscles [21]. SEC was defined as a dynamic smoke-like signal that swirls slowly in a circular pattern within the LA and LAA and has an optimal gain setting for distinguishing SEC from echoes due to excessive gain [21]. Clinical criteria have been established to grade SEC severity as mild, moderate, or severe. In this study, all grades of SEC were included in the SEC group [5].

### 2.4. Statistical Analysis

Statistical analyses were performed using SPSS (SPSS 22.0J, Chicago, U.S.A.). The data were expressed as means ± SD and categorical variables as percentages. The T test was used to compare continuous variables. The chi-squared test or Fisher's exact test was utilized for comparing discontinuous variables. Comparisons between baseline characteristics in the three groups (patients without thrombus versus patients with SEC and thrombus) were done using one-way analysis of variance (ANOVA) and least significant difference (LSD) test. Receiver operating curves (ROCs) were generated to define the cutoff values (the upper left corner of ROC is considered as the point of maximum sensitivity and specificity) of D-dimer, NT-proBNP, and RDW for the presence of LA/LAA thrombus in the study population. In addition, univariate and multivariate binary logistic regression analyses were performed to investigate independent correlates of LA/LAA thrombus. Variables with *p* < 0.05 in univariate analysis were included into multivariate regression analysis. Two-side *p* value <0.05 was considered to be statistically significant in all analyses.

## 3. Results

At baseline, there were 260 patients with NVAF who were on anticoagulation therapy, with an average age of 63.67 ± 9.39 years and 42% of these were women. The study included 84 (33%) patients without thrombus, 131 (50%) patients with LA/LAA SEC, and 45 (17%) patients with LA/LAA thrombus. Clinical and demographic characteristics of the study population are summarized in Table 1. Frequency of patients with paroxysmal AF was higher in the no thrombus group when compared with the SEC group and thrombus group (60.7% vs. 49.6% vs. 46.7%; *p* < 0.001). The frequency of patients with smoking history, drinking history, coronary heart disease after PCI, old myocardial infarction, hypertension, diabetes mellitus, stroke/TIA, and recurrent AF after ablation showed no significant differences between the no thrombus group and thrombus group. The mean CHA_2_DS_2_-VASc scores and the percentage of patients with CHA_2_DS_2_-VASc scores ≥2 among three groups showed no statistically significant differences. All 260 patients had warfarin, dabigatran, or rivaroxaban treatment. Only patients without LA/LAA thrombus had underwent AF ablation procedure, and none of the treated patients had thromboembolism.

Laboratory parameters of the study groups are compared in Table 2. Of all hematological parameters, erythrocyte count, RDW, and prothrombin time (PT) showed statistically significant differences, while platelet count, platelet distribution width (PDW), and fibrinogen (FBI) showed no significant differences among three groups. In the thrombus group, erythrocyte count (4.77 ± 0.51 vs. 4.60 ± 0.49 vs. 4.55 ± 0.45; *p*=0.047) and RDW (13.19 ± 0.91 vs. 12.96 ± 0.64 vs. 12.76 ± 0.52; *p*=0.002) levels were significantly higher when compared with the SEC group and no thrombus group, respectively. When patients with LA/LAA thrombus were compared with those without thrombus, the D-dimer levels (503.48 ± 674.72 vs. 454.85 ± 538.92 vs. 414.87 ± 426.15; *p*=0.671) showed no significant differences. However, the NT-proBNP levels (1152.34 ± 997.06 vs. 574.20 ± 548.28 vs. 373.91 ± 394.70; *p* < 0.001) were significantly higher in patients with thrombus in LA/LAA.

Echocardiographic parameters of the study groups are compared in Table 3. As expected, the LAD (44.77 ± 6.12 vs 42.04 ± 6.08 vs 38.02 ± 5.11; *p* < 0.001) was significantly higher in the thrombus group, and had lower ejection fraction (58.50 ± 6.70 vs 60.87 ± 6.40 vs 62.99 ± 5.75; *p*=0.001).

Multiple logistic regression analysis was performed to determine the effects of echocardiographic parameters (LAD, LVEF, and RVEDD), laboratory parameters (RBC, RDW, D-dimer, and NT-proBNP), and AF duration (paroxysmal AF) on the likelihood of patients with LA/LAA thrombus. Of the eight predictor variables, RDW (OR, 2.398; 95% CI: 1.075–5.349; *p*=0.033) and NT-proBNP (OR, 2.179; 95% CI: 1.191–3.987; *p*=0.012) showed statistically significant differences in Table 4.

**Figure 1:**
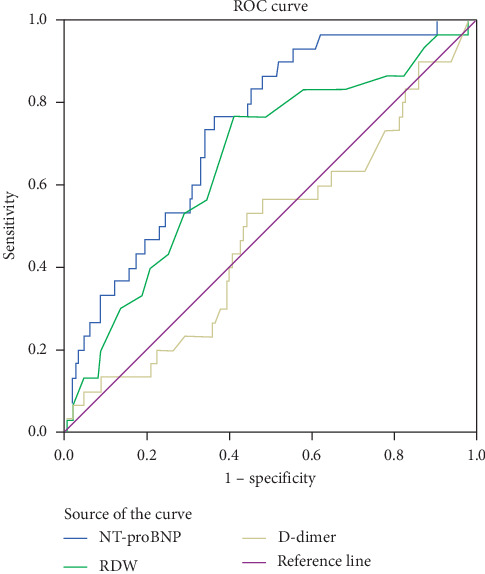
ROC curve for predicting LA/LAA thrombosis in patients with NVAF.

To determine the effect of RDW, D-dimer, and NT-proBNP on the presence of LA/LAA thrombus, the ROC curve analysis is performed in Table 5 and Figure 1. NT-proBNP and RDW were predictive of LA/LAA thrombus with an AUC of 0.749 (95% CI: 0.656–0.842; *p* < 0.001) and 0.666 (95% CI: 0.551–0.780; *p*=0.005) and were significantly higher than that of 0.446 of the D-dimer (95% CI: 0.319–0.576; *p*=0.062). Furthermore, NT-proBNP was superior to that of RDW. The cutoff value of RDW was predicted to be 12.95% in the presence of LA/LAA with a sensitivity of 75.0% and a specificity of 60.5%. When the cut-off value of NT-proBNP was 368.9 ng/L, the sensitivity was 85.7%, and the specificity was 54.6%.

**Figure 2:**
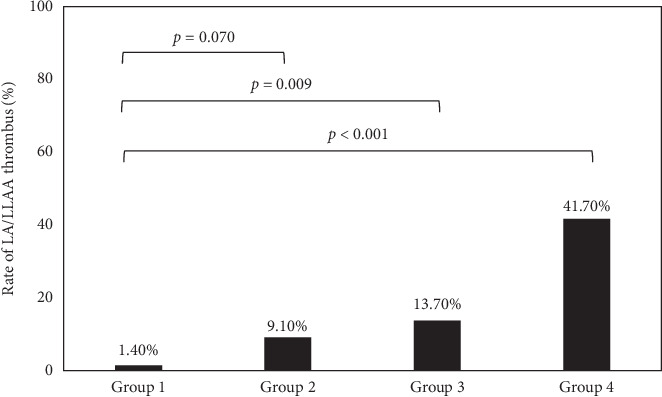
The rate of patients with LA/LAA thrombus.

In addition, all patients were divided into four groups based on the combination between RDW (cutoff value: 12.95%) and NT-proBNP levels (cutoff value: 368.9 ng/L): group 1 (RDW < 12.95% and NT-proBNP < 368.9 ng/L, *n* = 71), group 2 (RDW ≥ 12.95% and NT-proBNP < 368.9 ng/L, *n* = 44), group 3 (RDW < 12.95% and NT-proBNP ≥ 368.9 ng/L, *n* = 73), and group 4 (RDW ≥ 12.95% and NT-proBNP ≥ 368.9 ng/L, *n* = 72). The prevalence of LA/LAA thrombus in Figure 2 was 1.40, 9.10, 14.70, and 41.70% in groups 1, 2, 3, and 4, respectively. The rate of LA/LAA thrombus was the highest in the group 4.

## 4. Discussion

This study demonstrated that high levels of NT-proBNP and RDW showed significant and independent association with the presence of LA/LAA thrombus. The major findings of this study were as follows: firstly, the RDW and NT-proBNP levels were significantly higher in patients with thrombus than SEC patients and no thrombus patients; secondly, when all patients were divided into four groups based on the combination between RDW (cutoff value: 12.95%) and NT-proBNP levels (cutoff value: 368.9 ng/L), the rate of LA/LAA thrombus was the highest in the high RDW and NT-proBNP group; and thirdly, the D-dimer values failed to predict the LA/LAA thrombus in this statistical model.

The RE-LY biomarker study first reported that the levels of NT-proBNP showed correlation with the risk of thromboembolic events and cardiovascular mortality, increasing the risk with increased levels [15]. The results from the larger ARISTOTLE biomarker study (*n* = 14,892) were consistent with those of the previous findings of the RE-LY trial [8]. Besides, addition of NT-proBNP levels to the CHA_2_DS_2_VASc score improved the C-statistics from 0.62 to 0.65 (*p*=0.0009) for stroke or systemic embolism, providing evidence that the NT-proBNP levels might be a novel tool for improving stroke prediction in AF. However, there was conflicting data regarding the association of LAA thrombi and thromboembolic strokes/events. Kawabata et al. found no significant association between LAA thrombi and embolic events in patients with chronic NVAF [22]. Other studies suggested LAA thrombi as a precursor of systemic thromboembolism and stroke [1]. Thus, there were many studies that further explored the relationship between NT-proBNP and LA/LAA thrombosis. Most of the evidence suggested NT-proBNP as an independent predictor of LAA thrombus in patients with AF [22–24]. It is well known that NT-proBNP showed a significant increase in patients with heart failure. Therefore, patients with existing symptoms of severe heart failure were excluded in this study and the results showed significantly higher levels of NT-proBNP in the plasma of patients with LAA thrombus than in those without LAA thrombus. Therefore, our study supported that elevation of NT-proBNP levels acts as a strong predictor of LA/LAA thrombosis in NVAF patients.

RDW acted as a measure of the variability for circulating erythrocyte volume and was positively correlated with the risk of developing thromboembolic events and even thrombus formation in many clinical and epidemiological studies [6, 25, 26]. In this study, RDW was found to be an independent risk factor of thrombosis in patients with NVAF (OR, 2.398; *p*=0.033) and a moderate predictive value for thrombus formation was demonstrated, with an area under the RDW curve of 0.666 (*p*=0.005). However, it was still unclear whether this association indicates independent contribution of RBCs to thrombosis. But, a growing body of evidence from mechanistic studies suggested that RBCs might contribute to venous thrombus formation by increasing blood viscosity, aggregation, adherence to the vessel wall, promotion of thrombin generation, and increasing thrombus size [16, 17]. Regarding the relationship between RDW and AF thrombosis, several studies suggested that inflammation and oxidative stress played an important role in the occurrence and persistence of AF [6, 27–29]. The release of proinflammatory cytokines inhibited erythropoietin-induced erythrocyte maturation [27]. Oxidative stress affected the RDW levels by reducing red blood cell lifespan and hemolytic capacity. The RDW values showed an elevation in patients with increased levels of oxidative stress [28]. SEC was a prethrombotic state, which presented erythrocyte aggregation caused by interaction between the cells and fibrinogen. Erythrocyte irregularity led to erythrocyte aggregation, thus increasing the degree of SEC [30]. Therefore, we hypothesized that there might be a close relationship between the RDW levels and LA/LAA SEC and thrombosis in NVAF patients.

D-dimer was generally considered as a gold standard for the detection of coagulation and fibrinolysis [31]. Several studies demonstrated that D-dimer levels had a high negative predictive value with regard to thrombus formation in patients with NVAF [7, 11, 32–34]. Habara et al. showed that the D-dimer cutoff level of 1.15 *μ*g/mL had a negative predictive value of 97% in identifying the LAA thrombi [7]. You et al. found that only the D-dimer levels during the onset of stroke but not the baseline D-dimer levels acted as an independent risk factor of ischemic stroke [12, 35]. However, the ARISTOTLE trial proved that the baseline D-dimer levels were related to the rate of stroke/systemic embolism in patients with AF and increased the predictive value of clinical risk scores. This was consistent with the results of RE-LY trial [10, 11]. Combination of D-dimer levels and CHADS_2_ score improved the C-index from 0.646 to 0.655 in stroke or systemic embolism. But the D-dimer levels at baseline in the NOAC group was higher than that of the warfarin group (*p* < 0.0001) in the ARISTOTLE trial. Therefore, we speculated that anticoagulant therapy might weaken the predictive value of D-dimer in LA/LAA thrombus, hindering the emergence of D-dimer as an independent risk factor for multivariate analysis in this study. Small sample size might also lead to the failure of the correlation between baseline D-dimer levels and LA/LAA thrombus.

This was a single-center retrospective study with a small sample size, and the potential causal relationship could not be defined clearly. This was an observational study; there may be unknown confounders that were not adjusted for and may therefore bias the findings. Hence, it has become difficult to determine the significance of prognosis. Secondly, routine iron, vitamin B12, folic acid, C-reactive protein, and other factors were not tested in this study. Further, large-scale and prospective studies are needed to confirm the role of D-dimer, RDW, and NT-proBNP in predicting LA/LAA thrombus in patients with NVAF.

## 5. Conclusion

This study demonstrated a significant association between high levels of NT-proBNP and RDW in the presence of thrombosis in NVAF patients, whereas the D-dimer levels failed to act as an independent risk factor in LA/LAA thrombus.

## Figures and Tables

**Table 1:** Baseline characteristics of NVAF patients with or without thrombus and SEC.

Variable	No thrombus group (*n* = 84)	SEC group (*n* = 131)	Thrombus group (*n* = 45)	*p* value
Age (years)	62.58 ± 10.12	64.41 ± 8.42	63.56 ± 10.61	0.379
Male	51 (60.7)	74 (56.5)	27 (60)	0.807
Smoking history	18 (21.4)	36 (27.5)	17 (37.8)	0.139
Drinking history	13 (15.5)	15 (11.5)	10 (22.2)	0.203
History of coronary heart disease	37 (44.0)	47 (35.9)	16 (35.6)	0.441
After PCI	10 (11.9)	14 (10.7)	4 (8.9)	0.870
Old myocardial infarction	3 (3.6)	4 (3.1)	1 (2.2)	0.914
Hypertension	51 (60.7)	77 (58.8)	23 (51.1)	0.559
Diabetes mellitus	16 (19.0)	27 (20.6)	10 (22.2)	0.909
Stroke/TIA	11 (13.1)	18 (13.7)	19 (20)	0.527
Paroxysmal AF	66 (78.6)	65 (49.6)^*∗*^	23 (46.7)^*∗*^	<0.001
CHA_2_DS_2_-VASc score	2.20 ± 1.63	2.19 ± 1.60	2.20 ± 1.71	0.630
CHA_2_DS_2_-VASc score ≥2	53 (63.1)	81 (61.8)	26 (57.8)	0.835
Recurrent AF after ablation	8 (9.5)	11 (8.4)	2 (4.4)	0.590

AF = atrial fibrillation; TIA = transient ischemic attack; CHA_2_DS_2_-VASc = congestive heart failure, hypertension, age, diabetes mellitus, stroke, vascular disease, and sex.^*∗*^*p* < 0.05 vs. no thrombus group.

**Table 2:** Laboratory parameters and echocardiographic parameters among the three groups.

Variable	No thrombus group (*n* = 84)	SEC group (*n* = 131)	Thrombus group (*n* = 45)	*p* value
RBC (10^12^/L)	4.55 ± 0.45	4.60 ± 0.49	4.77 ± 0.51^*∗*^^§^	0.047
Hb (g/L)	142.49 ± 13.40	143.12 ± 15.16	145.75 ± 17.62	0.493
MCV (fL)	93.24 ± 4.03	93.30 ± 4.27	91.83 ± 6.23	0.165
MCH (pg)	31.37 ± 1.51	31.14 ± 1.53	30.59 ± 2.46^*∗*^	0.053
RDW (%)	12.76 ± 0.52	12.96 ± 0.64^*∗*^	13.19 ± 0.91^*∗*^^§^	0.002
Plt (10^9^/L)	214.83 ± 42.68	209.08 ± 47.77	224.20 ± 61.25	0.199
PCT	0.21 ± 0.04	0.20 ± 0.04	0.22 ± 0.05	0.321
PDW (%)	16.10 ± 0.63	16.12 ± 0.32	16.13 ± 0.30	0.910
PT (s)	12.43 ± 3.52	13.26 ± 4.17	14.54 ± 6.70^*∗*^	0.043
FIB (g/L)	2.80 ± 0.65	2.88 ± 0.74	2.84 ± 0.85	0.695
Urea (mmol/L)	5.77 ± 1.43	6.02 ± 1.93	6.02 ± 1.80	0.568
Cr (*μ*mol/L)	74.86 ± 24.32	76.06 ± 27.83	75.87 ± 19.64	0.943
CK-MB (ng/mL)	10.33 ± 5.16	11.89 ± 8.26	11.24 ± 4.83	0.282
TC (mmol/L)	4.76 ± 1.16	4.88 ± 1.03	4.88 ± 1.40	0.725
TG (mmol/L)	1.85 ± 1.45	2.04 ± 1.85	1.97 ± 1.62	0.701
HDLc (mmol/L)	1.14 ± 0.24	1.13 ± 0.28	1.12 ± 0.31	0.905
LDLc (mmol/L)	2.92 ± 0.93	3.03 ± 0.80	3.02 ± 1.01	0.665
D-dimer (ng/mL)	414.87 ± 426.15	454.85 ± 538.92	503.48 ± 674.72	0.671
cTnI (ng/mL)	0.004 ± 0.007	0.004 ± 0.006	0.005 ± 0.007	0.443
NT-proBNP (ng/L)	373.91 ± 394.70	574.20 ± 548.28^*∗*^	1152.34 ± 997.06^*∗*^^§^	<0.001

MCV = mean hemoglobin volume; MCH = mean hemoglobin content; RDW = red cell distribution width; Plt = platelet; PCT = lateletcrit; PDW = platelet distribution width; PT = prothrombin time; FIB = fibrinogen; urea = urea nitrogen; Cr = creatinine; CK-MB = creatine kinase isoenzyme; TC = cholesterol; TG = triglyceride; HDLc = high-density lipoprotein cholesterol; LDLc = low -density lipoprotein cholesterol; cTnI = cardiac troponin I; NT-proBNP = N-terminal pro-B-type natriuretic peptide.^*∗*^*p* < 0.05 vs no thrombus group.^§^*p* < 0.05 vs SEC group.

**Table 3:** Echocardiographic parameters among the three groups.

Variable	No thrombus group (*n* = 84)	SEC group (*n* = 131)	Thrombus group (*n* = 45)	*p* value
LVEDD (mm)	49.47 ± 7.27	49.63 ± 6.34	51.60 ± 6.87	0.203
LAD (mm)	38.02 ± 5.11	42.04 ± 6.08^*∗*^	44.77 ± 6.12^*∗*^^§^	<0.001
RVEDD (mm)	19.72 ± 2.29	20.88 ± 2.32^*∗*^	21.22 ± 2.67^*∗*^	0.001
LVEF (%)	62.99 ± 5.75	60.87 ± 6.40^*∗*^	58.50 ± 6.70^*∗*^^§^	0.001

LVEDD = left ventricular end-diastolic diameter; LAD = left atrial diameter; RVEDD = right ventricular end-diastolic diameter; LVEF = left atrial ejection fraction.^*∗*^*p* < 0.05 vs no thrombus group.^§^*p* < 0.05 vs SEC group.

**Table 4:** Multivariate logistic regression analysis of thrombus in LA/LAA.

Variable	*B*	SE	Wald *χ*^2^	*p* value	OR	95% CI
LVEF	−0.035	0.036	0.935	0.334	0.966	0.900–1.036
LAD	0.082	0.048	2.936	0.087	1.086	0.988–1.193
RVEDD	0.022	0.096	0.050	0.823	1.022	0.846–1.234
RBC	0.773	0.495	2.435	0.119	2.166	0.821–5.716
D-dimer	−0.001	0.000	1.573	0.210	0.999	0.998–1.000
RDW	0.875	0.409	4.565	0.033	2.398	1.075–5.349
Paroxysmal AF	0.316	0.549	0.331	0.565	1.371	0.468–4.018
LnNT-ProBNP	0.779	0.308	6.383	0.012	2.179	1.191–3.987

LAD = left atrial diameter; RVEDD = right ventricular end-diastolic diameter; LVEF = left atrial ejection fraction; RDW = red cell distribution width; LnNT-ProBNP =  logarithm of NT-proBNP with base e.

**Table 5:** ROC curve for predicting LA/LAA thrombosis in patients with NVAF.

Variable	Area under the curve	*p* value	95% CI
NT-proBNP	0.749	<0.001	0.656–0.842
RDW	0.666	0.005	0.551–0.780
D-dimer	0.446	0.062	0.345–0.587

## Data Availability

The data used to support the findings of this study are available from the corresponding author upon request.
